# ASSESSMENT OF THE GASTRO-JEJUNO-DUODENAL TRANSIT AFTER JEJUNAL POUCH INTERPOSITION

**DOI:** 10.1590/S0102-6720201500040003

**Published:** 2015

**Authors:** Alcino Lázaro da SILVA, Célio Geraldo de Oliveira GOMES

**Affiliations:** Clinics Hospital, Federal University of Minas Gerais, Belo Horizonte, MG, Brazil

**Keywords:** Gastrointestinal transit, Gastrectomy, Gastric emptying, Post-gastrectomy syndrome, Dumping syndrome

## Abstract

**Background:**

The jejunal pouch interposition between the gastric body and the duodenum after the gastrectomy, although not frequent in the surgical practice today, has been successfully employed for the prevention and treatment of the postgastrectomy syndromes. In the latter, it is included the dumping syndrome, which affects 13-58% of the patients who undergo gastrectomy.

**Aim:**

Retrospective assessment of the results of this procedure for the prevention of the dumping syndrome.

**Methods:**

Fourty patients were selected and treatetd surgically for peptic ulcer, between 1965 and 1970. Of these, 29 underwent vagotomy, antrectomy, gastrojejunalduodenostomy at the lesser curvature level, and the 11 remaining were submitted to vagotomy, antrectomy, gastrojejunal-duodenostomy at the greater curvature level. The gastro-jejuno-duodenal transit was assessed in the immediate or late postoperative with the contrasted study of the esophagus, stomach and duodenum. The clinical evolution was assessed according to the Visick grade.

**Results:**

Of the 40 patients, 28 were followed with the contrast evaluation in the late postoperative. Among those who were followed until the first month (n=22), 20 (90%) had slow gastro-jejuno-duodenal transit and in two (10%) the transit was normal. Among those who were followed after the first month (n=16), three (19%) and 13 (81%) had slow and normal gastric emptying, respectively. None had the contrasted exam compatible with the dumping syndrome. Among the 40 patients, 22 underwent postoperative clinical evaluation. Of these, 19 (86,5%) had excellent and good results (Visick 1 and 2, respectively).

**Conclusions:**

The jejunal pouch interposition showed to be a very effective surgical procedure for the prevention of the dumping syndrome in gastrectomized patients.

## Abstract (pt)

**Racional:**

A interposição de alça jejunal entre o corpo gástrico e o duodeno após a antrectomia, apesar de pouco frequente na prática cirúrgica atual, tem sido empregada com sucesso na prevenção e tratamento das síndromes pós-gastrectomias. Entre estas se inclui a síndrome de dumping, que acomete 13-58% dos pacientes gastrectomizados.

**Objetivo:**

Avaliação retrospectiva dos resultados desse procedimento na prevenção da síndrome de dumping.

**Métodos:**

Foram selecionados 40 pacientes todos encaminhados para tratamento cirúrgico de úlcera cloridropéptica entre 1965 e 1970. Destes, 29 foram submetidos à vagotomia, antrectomia, gastrojejunoduodenostomia no nível da pequena curvatura, e os 11 restantes à vagotomia, antrectomia, gastrojejunoduodenostomia no nível da grande curvatura. O trânsito gastrojejunoduodenal foi avaliado no pós-operatório imediato ou tardio por meio do estudo contrastado de esôfago, estômago e duodeno. A evolução clínica no pós-operatório foi avaliada segundo a classificação de Visick.

**Resultados:**

Dos 40 pacientes, 28 foram acompanhados com o estudo contrastado no pós-operatório tardio. Entre aqueles de até o 1º mês de pós-operatório (n=22), 20 (90%) apresentaram o trânsito gastrojejunoduodenal lento e dois (10%) tiveram o trânsito normal. Entre os que puderam ser acompanhados após o 1º mês (n=16), três (19%) e 13 (81%) mostraram o esvaziamento gástrico lento e normal, respectivamente. Nenhum apresentou o estudo contrastado compatível com a síndrome de dumping. Entre os 40 doentes, 22 foram submetidos à avaliação clínica pós-operatória. Destes, 19 (86,5%) apresentaram excelentes e bons resultados (Visick 1 e 2, respectivamente).

**Conclusões:**

A interposição de alça jejunal mostrou-se procedimento cirúrgico bastante eficaz na prevenção da síndrome de dumping em pacientes gastrectomizados.

## INTRODUCTION

This study was a development from a previous article published by Resende Alves^16 ^in 1965, where a segment of the proximal jejunum was interposed between the gastric stump and the duodenum. This was proposed to prevent dumping syndrome (DS) after combined vagotomy, antrectomy, and gastroduodenostomy of either the lesser or the greater gastric curvature when treating chlorhydropeptic ulcers.

Postgastrectomy syndromes include reflux gastritis and esophagitis, postvagotomy diarrhea, afferent and efferent loop syndromes, and DS. The last may affect up to 58% of gastrectomized patients in the postoperative phase^1^^,^ 3 ^,^ 8 ^,^ 12. These complications are treated by a number of surgical interventions, of which Roux-en-Y gastrojejunostomy is most often chosen. Other options are Billroth I gastroduodenostomy, Billroth II gastrojejunostomy, and, more rarely, the interposition of a pouch made from a jejunal loop between the stomach and the duodenum^6^.

The interposition of a jejunal pouch was first described by Henley^9^ in 1952, and later modified by Soupault-Bucaille. Although infrequent in today's surgical practice, it has been used with good results in preventing and treating postgastrectomy syndromes, particularly DS.

This study aims to present the results of interposing a segment of the proximal jejunum between the gastric stump and the duodenum in treating chlorhydropeptic ulcer, analyzing gastric emptying and late postoperative symptoms. 

## METHODS

**FIGURE 1:**
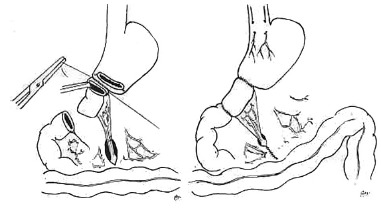
- Left: termino-terminal anastomosis of the cranial end of the isolated jejunal loop segment to the stomach's lesser curvature. Right: completed surgery, with vagotomy, antrectomy, and interposition of the loop segment between the stomach and the duodenum.

In total, 40 patients were selected. All underwent surgical treatment of either gastric or duodenal chlorhydropeptic ulcer at the Clinics Hospital of the Federal University of Minas Gerais, Belo Horizonte, Minas Gerais, Brazil, between 1965 and 1970. Of 40, 29 of the patients underwent combined vagotomy, antrectomy, and gastrojejunalduodenostomy at the lesser curvature level; for the remaining 11, the gastrojejunalduodenostomy was at the greater curvature level. In all cases, a jejunal loop segment approximately 20 cm long was interposed isoperistaltically (Figure 1).

Gastrojejunoduodenal transit was assessed in both the early and the late postoperative period by a contrasted radiological examination of the esophagus, stomach, and duodenum. Postoperative clinical evolution was assessed in 22 patients according to the Visick grade, and postoperative complications were recorded as well.

## RESULTS

Of the 40 selected patients, 32 (80%) were men, and eight (20%) were women. Mean age was 39.1 years; 28 patients were followed up with a postoperative contrasted examination, and 22 were clinically evaluated after surgery according to the Visick grade.

**FIGURE 2:**
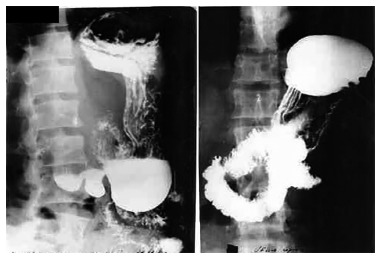
- Left: preoperative examination with partial stenosis of the duodenal bulb; right: interposed pouch and duodenum filled with barium

**FIGURE 3:**
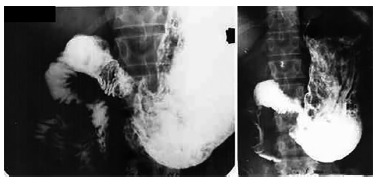
- Left: barium-filled stomach and small intestine; right: emptying occurring at a normal pace without excess

Of those who had a contrasted examination performed up to one month after surgery (n=22), 20 (90%) were shown to have slow transit, and two (10%) had normal transit. Twelve could not be followed up; however, six new patients were followed up after the first month. Of those who were followed up after the first month (n=16), three (19%) had slow transit, and 13 (81%) had normal transit; in these cases, the mean follow-up period was 19.7 months. No patient had a contrasted study compatible with DS (Figures 2 and 3).

Of the 22 patients who were clinically evaluated after surgery, 19 (85%) had excellent (Visick 1) to good (Visick 2) results, two (9%) had moderate symptoms (Visick 3), and one (4.5%) had a bad outcome (Visick 4). This latter patient was later operated again for an ulcer that had perforated the interposed pouch down to the head of the pancreas. These cases were followed up for an average of 13.4 months.

One patient died in the early postoperative period because of a duodenal fistula with necrosis of the interposed pouch; one patient had an ulcer of the pouch and three had wall abscesses. Other complications included light or moderate hemorrhage (n=2), pneumonia (n=1), narrowed anastomosis (n=1), jejunojejunal invagination (n=1), and partial dehiscence of a skin suture (n=1).

## DISCUSSION

Early diagnosis and more effective treatment of gastric cancer and other stomach diseases have made survival periods after gastrectomy increasingly longer. Although tumors can be controlled locally or regionally, the aim must be not only to cure such patients but also to improve their postoperative quality of life, as they often suffer from postgastrectomy syndrome^10^.

Nowadays, chlorhydropeptic ulcers are rarely operated because medication is effective to treat the disease^5^. In the few cases where surgical treatment is chosen, DS may occur, as it did in the old times, particularly after gastrojejunal anastomosis^15^.

DS occurs more frequently early in the postoperative period, but it can also occur at later stages. In most cases, it can be controlled by diet. However, in some cases symptoms are either refractory or severe enough to make normal everyday activities impossible. Surgical treatment is suggested for such patients^18^.

Surgeons who do not use the duodenum for the restored transit accept Roux-en-Y gastrojejunostomy as a method to prevent postgastrectomy syndrome. It is particularly effective when treating alkaline-reflux gastritis and esophagitis^18^^,^ 19. However, it correlates with adverse reactions such as abdominal distension and pain, gastric fullness, nausea, and vomiting. These symptoms characterize the Roux-en-Y stasis syndrome, which can affect 10%-50% of patients^2^^,^ 7.

Because Billroth II reconstruction does not preserve the pylorus and the duodenum, it causes fast gastric emptying, and DS occurs in up to 50% of patients in the early postoperative period^6^.

In contrast, Billroth I reconstruction tends to cause DS less often, because it preserves duodenal transit. It is also a technically simpler and quicker procedure, but as noted by Morii *et al.* 12 *,* it has a greater incidence of postoperative reflux gastritis and DS than isoperistaltic jejunal pouch interposition. In addition, other authors have had good results using jejunal pouch interposition to prevent and treat DS^4^^,^ 13_._

This technique's efficacy, however, appears to depend on the length of the interposed jejunal pouch and on whether isoperistaltic or anisoperistaltic interposition was used, among other factors. Sawyers *et al.* 18 demonstrated anisoperistaltic interposition to be superior to isoperistaltic, with good and excellent results in 94% and 20% of patients, respectively. Morii *et al.* 12, in contrast, had 90% success in treating DS with isoperistaltic interposition.

The ideal length of the interposed pouch is also controversial. Some authors obtained the best results with a 10 cm pouch^4^^,^ 17 ^,^ 18. Using longer segments, particularly longer than 20 cm, appears to correlate with a smaller incidence of reflux gastritis^2^. On the other hand, in theory, it would tend to cause more stasis of the gastric contents. In the current study, where loop segments approximately 20 cm long were used, 90% of patients evaluated up to one month after surgery had slow gastric emptying, as shown by the findings from the contrasted examination of the esophagus, stomach, and duodenum. When that assessment was made after the first month, the percentage dropped to 19%. Moreover, of the eight patients who initially showed slow transit in the contrasted examination during the first month and could be followed up later, seven progressed to normal transit. These findings suggest a probable temporal adaptation between the remaining gastric stump after vagotomy and the interposed jejunal pouch in restoring the physiological gastrointestinal transit^11^^,^ 14.

Despite the mutilation caused by gastrectomy, DS was less frequent in these patients because antrectomy is more conservative and protected by vagotomy. This unpredictable complication has a small incidence in combined vagotomy, antrectomy, and gastrojejunalduodenostomy at the lesser curvature level^11^, but to avoid it, was chosen to interpose a jejunum segment between the gastric stump and the duodenum. Authors experience shows that neither DS nor metabolic or nutritional disturbances happen^12^.

## CONCLUSION

The jejunal pouch interposition showed to be a very effective surgical procedure for the prevention of the dumping syndrome in gastrectomized patients.
